# Strongyloidiasis Diagnosed by ELISA in Brussels: A Bicentric Retrospective Study

**DOI:** 10.3390/microorganisms14081781

**Published:** 2026-08-12

**Authors:** Juliette Becu, Charlotte Martin, Sigi Van den Wijngaert, Delphine Martiny, Carine Truyens, Maya Hites

**Affiliations:** 1 Clinic of Infectious Diseases, Hôpital Universitaire de Bruxelles (HUB), Université Libre de Bruxelles (ULB), 1070 Brussels, Belgium; ju.becu@hotmail.be; 2 Department of Infectious Diseases, Centre Hospitalier Universitaire Saint-Pierre, Université Libre de Bruxelles (ULB), 1000 Brussels, Belgium; 3 Department of Microbiology, Laboratoire Hospitalier Universitaire de Bruxelles-Universitair Laboratorium Brussel (LHUB-ULB), 1000 Brussels, Belgium; sigi.vandenwijngaert@lhub-ulb.be (S.V.d.W.);; 4 Faculty of Medicine and Pharmacy, University of Mons (UMONS), 7000 Mons, Belgium; 5 Laboratory of Parasitology, Faculty of Medicine, ULB Center for Research in Immunology (UCRI), Université Libre de Bruxelles (ULB), 1070 Brussels, Belgium; carine.truyens@ulb.be

**Keywords:** *Strongyloides stercoralis*, strongyloidiasis, eosinophilia, migrants, travelers, serology, Belgium

## Abstract

Despite a significant population of migrants from endemic countries and of Europeans traveling to these regions, no Belgian study and few European studies have reported on strongyloidiasis. This retrospective study, conducted between January 2012 and December 2021, aimed to compare demographic data, clinical characteristics and management appropriateness of adult patients from Hôpital Universitaire de Bruxelles-Erasme and Centre Hospitalier Universitaire Saint-Pierre who had a positive serology for *Strongyloides stercoralis*. Serology results were classified as weakly positive (WP) or positive (P), and the two groups were compared. Qualitative and continuous data were expressed as numbers and medians, respectively, and compared using Fisher’s exact or Wilcoxon–Mann–Whitney tests, with a *p*-value < 0.05 considered statistically significant. A total of 358 patients (50% females) were included, with a median age of 52 (IQR: 40–63): 225 (62.8%) had WP and 133 (37.2%) had P results. Overall, 217/358 (60.6%) of the patients had at least one comorbidity, and 52/358 (14.5%) had an immunosuppressive factor. In total, 120/358 (33.5%) were symptomatic and 160/352 (45.5%) had blood eosinophilia. Only 51/358 (14.2%) benefitted from appropriate clinical management. Severe complications occurred in 17/358 (4.7%) patients, with two deaths (0.6%). This first Belgian study about strongyloidiasis shows that, despite low rates of severe complications and mortality, significant treatment delays were observed, requiring future improvement and monitoring.

## 1. Introduction

Strongyloidiasis is a geo-helminthiasis transmitted by direct contact between the skin and soil contaminated with *Strongyloides stercoralis* (*S. stercoralis*). This parasitosis is endemic in rural areas of tropical and subtropical regions, mainly in Sub-Saharan Africa, South America and South-East Asia. In January 2023, the World Health Organization (WHO) estimated global prevalence at around 600 million infected individuals [1]. In Europe, most reported cases are among migrants and travelers from endemic areas, but the exact number of people affected remains unknown [2,3].

The clinical spectrum of strongyloidiasis ranges from asymptomatic infection in most cases to acute symptomatic disease. Symptoms, when present, reflect the parasite’s migration pathway and are therefore dermatological, respiratory and digestive [4,5]. Through its ability to cause autoinfection, *S. stercoralis* can persist for decades, and potentially lifelong, in the human host [5,6], which may occasionally lead to a hyperinfection syndrome (HIS) in immunocompromised patients. This potentially fatal complication without treatment requires preventive screening before initiating immunosuppressive therapy [6,7,8,9,10].

According to the 2018 World Gastroenterology Organization recommendations, the “gold standard” diagnosis of strongyloidiasis includes serology plus parasitological stool examination (PSE). All individuals infected with *S. stercoralis* should be treated, even asymptomatic cases, due to the risk of hyperinfection and the self-infecting cycle of the parasite. The recommended first-choice treatment is a single dose of ivermectin at 200 μg/kg [8].

Despite the presence of a significant population of migrants from endemic countries and of Europeans traveling to these regions, no Belgian study and few European studies have been published to date about strongyloidiasis [11,12,13,14]. We carried out a retrospective study of patients with positive *S. stercoralis* serology at the Hôpital Universitaire de Bruxelles-Erasme (HUB-Erasme) and the Centre Hospitalier Universitaire Saint-Pierre (CHU Saint-Pierre). The main objective of our study was to describe the demographic characteristics, clinical presentation, and outcome of the patients and to compare these data according to the serological result. A second objective was to assess the appropriateness of clinical management in line with current recommendations.

## 2. Materials and Methods

### 2.1. General Principles and Serological Tests

We conducted a retrospective bicentric descriptive study of adults with positive serology for *S. stercoralis* cared for at HUB-Erasme (858 beds) or CHU Saint-Pierre (582 beds) from 1 January 2012 to 31 December 2021.

The patients included were identified via a list of those with a positive serology for *S. stercoralis* provided by each of the parasitology laboratories of the two hospitals. From this list were excluded: patients aged <18 years, those followed in other institutions and those with incomplete clinical files. Each patient was included only once, even in the case of repeated positive serologies over the ten-year study period.

Serological diagnosis of *S. stercoralis* was performed in both hospitals using indirect enzyme-linked immunosorbent assays (ELISAs) [15], including an in-house assay and the commercial SCIMEDX STRONGY-96 ELISA kit (SciMedx Corporation, Denville, NJ, USA). Both assays were based on a *Strongyloides ratti* antigen. Interpretation of results was as follows: i < 20, negative; 20 ≤ i < 30, very weakly positive; 30 ≤ i < 70, weakly positive; and i ≥ 70, positive (details in Appendix A).

### 2.2. Data Collection and Definitions (Further Details in Appendix B)

For each patient, we collected demographic characteristics, reasons for serological testing, comorbidities, history of strongyloidiasis, substance use, immunosuppressive factors (IF), clinical presentation, results from laboratory tests and digestive endoscopy, treatment, outcome and finally, the appropriateness of clinical management.

Countries were classified into non-endemic (prevalence < 5%), endemic (prevalence ≥ 5–10%) and hyperendemic (prevalence > 10%) for *S. stercoralis* [16,17]. We grouped together patients who had stayed in an endemic or hyperendemic region (EHR) during their childhood and/or while traveling.

The treatment delay (TD) was calculated as the time between the date of the first symptom of strongyloidiasis or the first documented eosinophilia and the administration of the first treatment. When both dates (symptoms and eosinophilia) were available, the longest delay was retained.

We defined three outcome categories for our cohort: favorable, unfavorable and unknown. A favorable outcome was defined by normalization of eosinophil count, resolution of symptoms attributed to *S. stercoralis*, a decrease in blood antibody levels against *S. stercoralis*, a negative PSE and/or the patient being alive and asymptomatic at follow-up. An unfavorable outcome included patients with persistent symptoms, those with eosinophilia persisting more than one year after a positive serodiagnosis without treatment, or more than one year after the last treatment received (in the absence of another known cause of eosinophilia) [18,19], as well as individuals who potentially died from *S. stercoralis* within one year of serodiagnosis. Severe complications such as bacterial infections and organ failure were also recorded. An unknown outcome referred to patients lost to follow-up.

Finally, we assessed the appropriateness of management. Management was considered inappropriate if any of the following criteria were not met: absence of a PSE in addition to serology, no encoded treatment for *S. stercoralis*, no repeated treatment in immunocompromised patients or in the presence of persistent symptoms, no serological follow-up 6 to 12 months after treatment and/or no eosinophil count monitoring at 12 months [8,18,20,21,22].

### 2.3. Statistical Analysis

Descriptive statistics were used to present the results. Qualitative variables were expressed as absolute numbers and percentages (%), and continuous variables were reported as medians with interquartile ranges. All the data were compared according to serological results, forming two groups: the weakly positive (WP) group (patients with very weakly positive or weakly positive serology), and the positive (P) group (patients with positive serology). Regarding outcomes, we analyzed them in relation to therapeutic management by comparing the untreated (NT) group (patients who received no treatment or for whom treatment status was unknown) with the treated (T) group.

Fisher’s exact test and the Wilcoxon–Mann–Whitney non-parametric test were used to compare qualitative and continuous variables, respectively.

Data were collected using Research Electronic Data Capture (REDCap; Vanderbilt University, Nashville, TN, USA). Statistical significance was defined as a two-tailed *p*-value of less than 0.05. Analyses were performed using SAS software version 9.4 (SAS Institute Inc., Cary, NC, USA).

The study was approved by the ethics committees of the HUB-Erasme (P2022/485) and CHU Saint-Pierre (CE/22-12-18).

## 3. Results

### 3.1. Patient Selection

**Figure 1:**
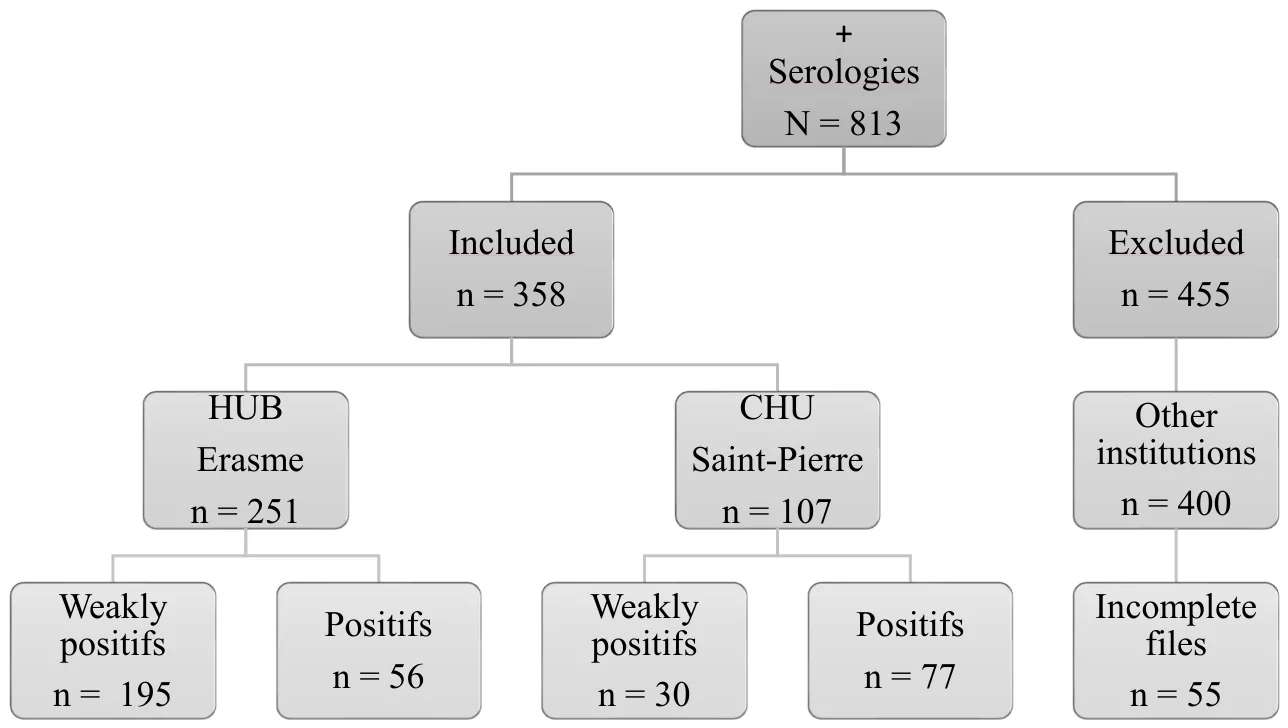
Flowchart of patient selection. Identification of eligible patients, reasons for applied exclusion criteria, and final study cohort.

A total of 813 adults with a positive serology for *S. stercoralis* were identified between 2012 and 2021. Among them, 455 patients were excluded, of whom 400 were followed in other institutions, and 55 had incomplete files. The remaining 358 patients were included, with 251 from HUB-Erasme and 107 from CHU Saint-Pierre. In total, 225 patients belonged to the WP group (52 with very WP and 173 WP serology), and 133 to the P group (Figure 1).

### 3.2. General Characteristics

Table 1 shows a balanced distribution between men and women [181/358 (50.6%)], with a median age of 52 years [40; 63]. Most patients were non-Caucasian [203/358 (56.7%)], with Sub-Saharan Africans representing the most frequent group [105/358 (29.3%)]. Compared to the WP group, the P group included a significantly higher proportion of non-Caucasian patients [104/225 (46.2%) vs. 99/133 (74.4%); *p* < 0.0001], with significantly higher proportions of Sub-Saharan Africans, Asians, and Hispanics. Regarding childhood and travel history, most patients [245/265 (92.4%)] had stayed in an EHR, with a significantly higher proportion in the P than in the WP group [112/116 (96.6%) vs. 133/149 (89.3%); *p* = 0.0335].

In the entire cohort, the main reason for serological testing were suggestive symptoms and signs [188/358 (52.5%)] (with a significantly higher proportion of WP group patients compared to P group patients [144/225 (64.0%) vs. 44/133 (33.1%); *p* < 0.0001]), followed by blood eosinophilia [131/358 (36.6%)] (with a significantly higher proportion of P group patients compared to WP group patients [83/133 (62.4%) vs. 48/225 (21.3%); *p* < 0.0001]) (Table 1).

Table 2 shows that 217/358 (60.6%) of the patients had at least one comorbidity, with arterial hypertension (AHT), diabetes, and chronic infectious diseases (CID) being the most common.

### 3.3. Clinical Presentation

**Figure 2:**
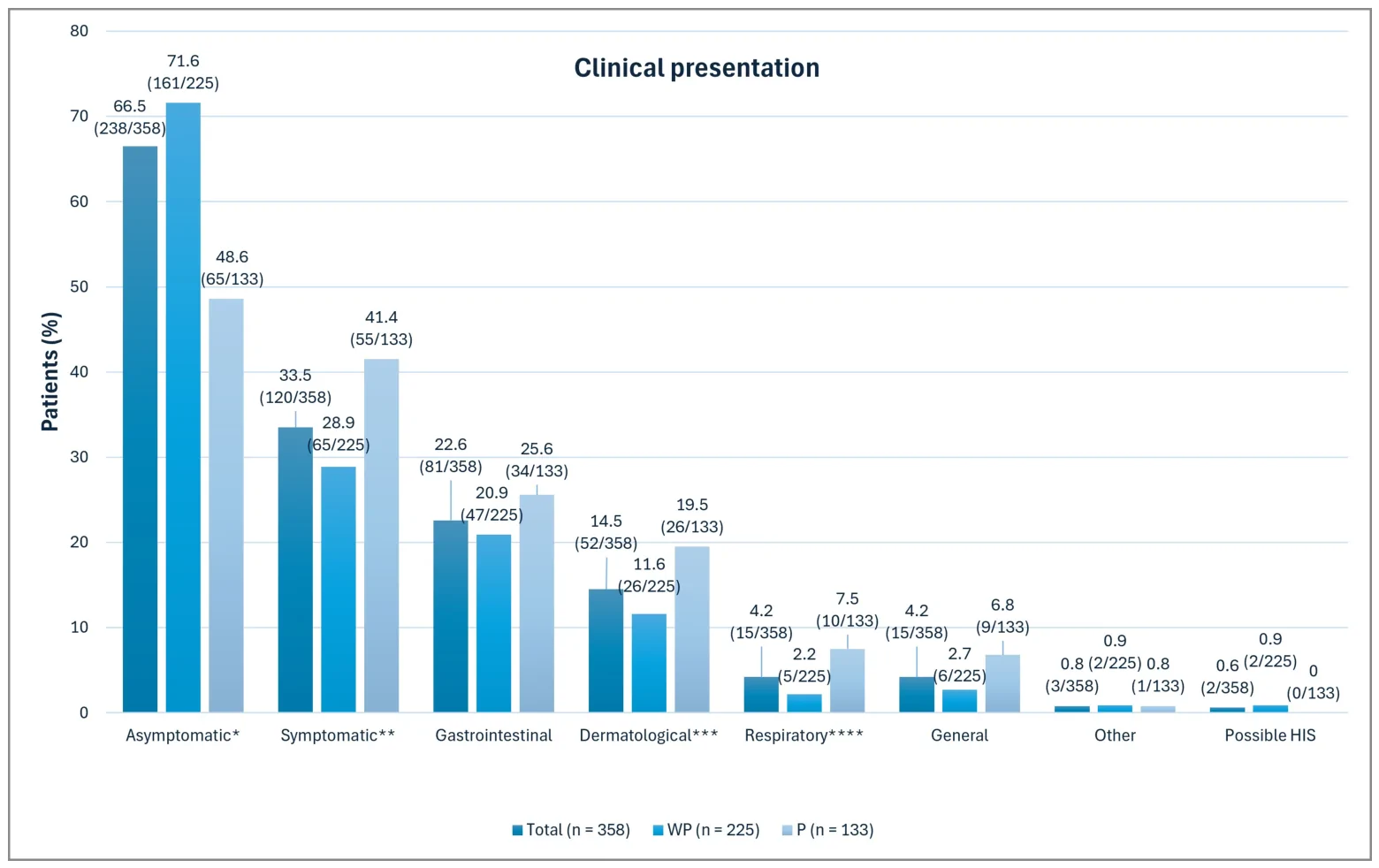
Clinical manifestations in patients with weakly positive (WP) and positive (P) *S. stercoralis* serology. * Significant difference between WP and P groups (161/225 (71.6%) versus 65/133 (48.6%); ***p*** < **0.0001**).** Significant difference between WP and P groups (65/225 (28.9%) versus 55/133 (41.4%); ***p*** = **0.0203**). *** Significant difference between WP and P groups (26/225 (11.6%) versus 26/133 (19.6%); ***p*** = **0.0440**). **** Significant difference between WP and P groups (5/225 (2.2%) versus 10/133 (7.5%); ***p*** = **0.0258**). Qualitative variables are expressed as absolute numbers and percentages (%). The statistical significance threshold was set at 0.05. Significant *p*-values are shown **in bold.** Abbreviations: HIS = hyperinfection syndrome; WP = weakly positive; P = positive.

Although serology was requested for suggestive symptoms for 188 patients, only 120/358 (33.5%) had symptoms that could not be attributed to other diseases, with a significantly higher proportion in the P group than in the WP group [55/133 (41.4%) vs. 65/225 (28.9%); *p* = 0.0203], as shown in Figure 2. The main symptoms were gastrointestinal and dermatological. Two probable cases of HIS were reported in the WP group.

**Figure A1:**
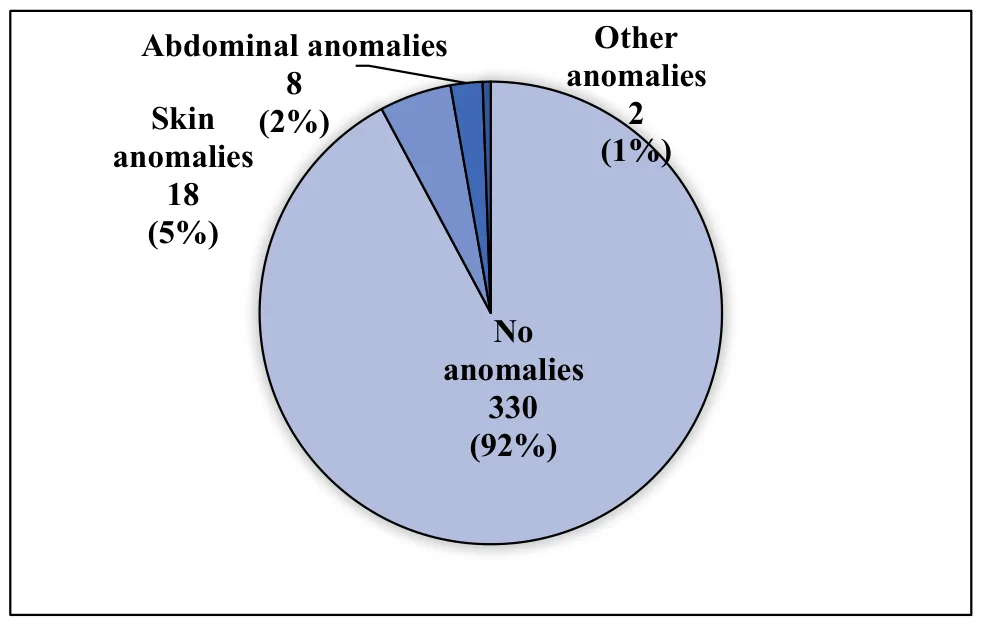
Physical examination of the cohort (n = 358).

Overall, 28/358 patients (7.8%) showed physical signs attributed to *S. stercoralis*, mostly cutaneous, with no significant difference between the two groups (P vs. WP) (Figure A1).

### 3.4. Laboratory Tests and Digestive Endoscopy

Blood eosinophilia was observed in 160 out of 352 patients (45.5%), with a significantly higher proportion in the P group than in the WP group [93/130 (71.5%) vs. 67/222 (30.2%); *p* < 0.0001]. Among patients tested for other parasitic infections, 133/319 (41.7%) had positive serology for other helminths, with a significantly higher rate in the P group than in the WP group [75/123 (60.6%) vs. 58/196 (29.6%); *p* < 0.0001]. Across the cohort, 240 parasitological stool examinations (PSE) were performed, with 5 testing positive for *S. stercoralis* and 39 for other parasites. Forty-five parasitological examinations (PE) of biological fluids were conducted, all of which were negative. Digestive endoscopy was performed in 136/358 patients (38.0%), with 46/136 (33.8%) conducted without a PSE. Digestive biopsies (DB) were performed in 107/358 patients (29.9%) and identified *S. stercoralis* in four cases, three of which were in the P group (Table A1).

### 3.5. Therapeutic Management

In total, 197/358 patients (55.0%) were treated for *S. stercoralis*, of whom 173/197 (87.8%) received ivermectin; among ivermectin-treated patients, 64/173 (37.0%) received a single dose. Treatment was administered significantly more often in the P group than the WP group [99/133 (74.4%) vs. 98/225 (43.6%); *p* < 0.0001] (Table A2).

**Figure 3:**
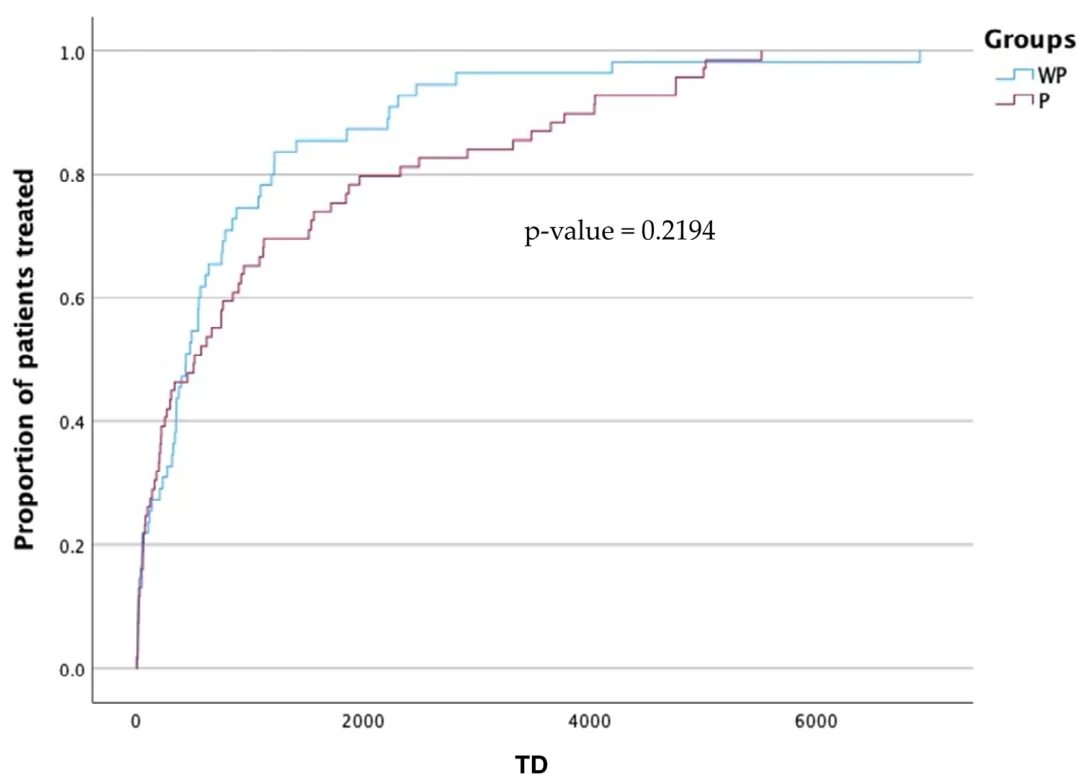
Cumulative distribution curve of treatment delay in patients with weakly positive (WP) and positive (P) *S. stercoralis* serology (n = 124). Abbreviations: WP = weakly positive; P = positive; TD = treatment delay (number of days between the date of first symptom or first documented eosinophilia and the date of treatment).

Figure 3 shows the cumulative distribution curve of treatment delay (TD) over time according to serological group. The median TD was 478 days [111–1217], with no significant difference between the two groups (P vs. WP). This analysis was based on 124 patients for whom TD could be calculated from available dates of the first symptom or first documented eosinophilia and treatment initiation. Patients with missing dates for either variable were therefore not included in this analysis.

### 3.6. Outcome and Appropriateness of Clinical Management

Table 3a,b show that, overall, 244/358 (68.1%), 40/358 (11.2%), and 74/358 (20.7%) patients had favorable, unfavorable, and lost-to-follow-up outcomes, respectively. A significantly higher proportion of treated patients (T) had a favorable outcome compared to non-treated patients (NT) [158/197 (80.2%) vs. 86/161 (53.4%); *p* < 0.0001]. Among NT patients with favorable outcomes, a significantly higher proportion belonged to the WP group compared to the P group [74/127 (58.3%) vs. 12/34 (35.3%); *p* = 0.0205]. Two deaths potentially attributable to *S. stercoralis* were observed in the NT group, corresponding to the two probable HIS cases, both of whom died from septic shock due to Gram-negative Bacilli bacteremia. A total of 17/358 patients (4.7%) experienced severe complications, with no significant difference between the two groups.

Across the cohort, 51/358 patients (14.2%) received appropriate clinical management, with a significantly higher proportion in the P group than in the WP group [33/133 (24.8%) vs. 18/225 (8.0%); p < 0.0001]. Among unmet criteria, lack of serological follow-up [273/358 (76.3%)] and absence of treatment [161/358 (45.0%)] were the most frequent, with significantly more cases in WP patients than in P patients, [185/225 (82.2%) vs. 88/133 (66.2%); p = 0.0008] and [127/225 (56.4%) vs. 34/133 (25.6%); p < 0.0001], respectively.

## 4. Discussion

This retrospective 10-year study includes 358 patients from HUB-Erasme and CHU Saint-Pierre hospitals in Brussels, Belgium, all with positive serology for *S. stercoralis*; among them, 225 were weakly positive (WP) and 133 were positive (P). The cohort comprised a gender-balanced population with a median age in the fifties, predominantly non-Caucasian, and with a history of travel to or childhood in an EHR. Despite 60% of patients presenting at least one comorbidity, clinical manifestations were generally mild, and the complication rate remained low (<5%). However, the management of strongyloidiasis remained suboptimal, with only two-thirds of patients undergoing stool examination, half receiving treatment, and a median treatment delay of 16 months. These findings highlight that, despite generally mild presentations, delayed recognition and insufficient treatment are important gaps in care in non-endemic settings.

Stratified analysis by serological group (WP vs. P) supported the hypothesis that higher antibody levels are associated with more active infection. Patients with P serology had twice as many symptoms, cases of blood eosinophilia, and positive digestive biopsies for *S. stercoralis* compared to WP patients. This higher clinical and biological burden likely contributed to more intensive management in the P group, including more frequent treatments, repeated treatments in immunocompromised individuals, and serological follow-up. However, the higher proportion of favorable outcomes without treatment observed in the WP group compared with the P group should not justify withholding treatment, as a prospective Australian study by Hays et al., involving 259 patients, demonstrated the benefit of treating weakly seropositive patients in high-prevalence settings [23], in line with WHO recommendations to treat all infected individuals regardless of antibody titer [8]. These findings suggest that antibody levels may reflect disease activity but should not determine treatment decisions alone.

Comparing our cohort to other retrospective studies in non-endemic regions (sample sizes ranging from 33 to 452 patients) [11,12,13,14,19,24], similar findings were observed regarding reasons for testing (symptoms, eosinophilia, screening), prior stays in EHR, proportion of immunosuppressed patients, and main symptom types (gastrointestinal and cutaneous). However, other studies report higher rates of symptomatic patients (36–75%) [11,12,13,14,19,24], blood eosinophilia (64–90%) [11,12,13,19,24], and HIS (1.3–15%) [11,12,24]. The frequency of symptoms may vary due to their intermittent and non-specific nature [19]. Blood eosinophilia correlates well with strongyloidiasis [14], although its intermittent presentation and differing definitions across studies limit its value as a screening tool [19,22]. In our study, only two probable HIS cases were identified, though some may have been missed due to false-negative serology [25] and limited awareness among Belgian physicians. These findings emphasize the need for increased clinical vigilance among patients at risk, particularly before immunosuppressive therapy.

Regarding diagnostic procedures, over one-third of patients underwent invasive tests with low diagnostic yield, and more than 30% of these had no prior stool examination. Detection of *S. stercoralis* in only 5 stool samples highlights the test’s low sensitivity: an inverse relationship between microscopic evidence and antibody presence has been described [13]. Improving awareness of appropriate diagnostic pathways may therefore help reduce unnecessary invasive procedures and delays in diagnosis.

Despite inappropriate management, two-thirds of the cohort had a favorable outcome. The 30% higher rate of favorable outcome among treated (T) versus non-treated (NT) patients supports the use of ivermectin, a safe and inexpensive drug. The long treatment delay, possibly attributable to mild clinical manifestations [9], may lead to more severe complications [26,27], and likely reflects delayed recognition of the infection. Earlier diagnosis and treatment initiation remain essential, especially in immunosuppressed patients at risk of severe disease.

Several studies, including ours, have shown that many patients seropositive for *S. stercoralis* also test positive for other helminths, suggesting either true co-infections or cross-reactivity [10,11,12,13,14,24]. In our cohort, most individuals seropositive for other helminths belonged to the P group, supporting true *S. stercoralis* infection. Differentiating between true infection and co-infection remains challenging and typically requires stool examination. However, first-line treatment with ivermectin provides broad-spectrum antiparasitic coverage.

This study is limited by its retrospective nature and missing data. As strongyloidiasis is often asymptomatic and only a small proportion of our cohort was screened, our results may not fully reflect the infected population in Brussels. However, its bicentric design over a 10-year period, large sample size, and inclusion of many at-risk individuals (migrants and immunosuppressed patients) are notable strengths. Another limitation is that patients diagnosed solely by stool analysis were not included. Nevertheless, given the higher sensitivity of serology, we likely detected most infections. Finally, standardized international criteria for appropriate infection management are still lacking.

## 5. Conclusions

This first study on strongyloidiasis in Belgium offers valuable information on the characteristics and management of patients with a positive serology for *S. stercoralis* in Brussels. Despite low rates of severe complications and mortality, treatment delays are excessive, and management remains suboptimal. With travel, migration, climate change and growing use of immunosuppressants, the prevalence and complications of strongyloidiasis are likely to rise in non-endemic areas. Therefore, prospective systematic screening of patients from endemic areas and medical education would enhance awareness and care in Belgium.

## Figures and Tables

**Table 1:** Comparison of demographic characteristics and reasons for *S. stercoralis* serological testing between the weakly positive (WP) and positive (P) groups.

Characteristics	Total (%)	WP (%)	P (%)	*p*-Value
**Number (% total)**	358 (100.0)	225 (62.8)	133 (37.2)	
**Age**	52 [40; 63]	50 [38; 60]	54 [44; 65]	**0.0111**
**Women**	181 (50.6)	122 (54.2)	59 (44.4)	0.0804
**Ethnicity**				
Caucasian	155 (43.3)	121 (53.8)	34 (25.6)	**<0.0001**
Sub-Saharan African	105 (29.3)	48 (21.3)	57 (42.9)	**<0.0001**
North African	54 (15.1)	40 (17.8)	14 (10.5)	0.0682
Asian	29 (8.1)	13 (5.8)	16 (12.0)	**0.0448**
Hispanic	15 (4.2)	3 (1.3)	12 (9.0)	**0.0007**
**Childhood and travel**				
With known data	265/358 (74.0)	149/225 (66.2)	116/133 (87.2)	**<0.0001**
Childhood and/or travel in EHR	245/265 (92.4)	133/149 (89.3)	112/116 (96.6)	**0.0335**
Neither childhood nor travel in EHR	20/265 (7.6)	16/149 (10.7)	4/116 (3.4)	**0.0335**
**Reasons for serological testing**				
Symptoms and suggestive signs	188 (52.5)	144 (64.0)	44 (33.1)	**<0.0001**
Blood eosinophilia	131 (36.6)	48 (21.3)	83 (62.4)	**<0.0001**
Screening	65 (18.2)	43 (19.1)	22 (16.5)	0.5734
NIC patient	33 (9.2)	19 (8.4)	14 (10.5)	0.5717
Pre-IS treatment	23 (6.4)	17 (7.6)	6 (4.5)	0.3724
IC patient	9 (2.5)	7 (3.1)	2 (1.5)	0.4935
History of parasitosis	34 (9.5)	30 (13.3)	4 (3.0)	**0.0012**
Other reason	26 (7.3)	16 (7.1)	10 (7.5)	1
Unknown	11 (3.1)	6 (2.7)	5 (3.8)	0.5454

Ethnicity categories were mutually exclusive and were assigned based on the demographic information available in the medical records. Non-Caucasian included Sub-Saharan African, North African, Asian, and Hispanic categories. Qualitative variables are expressed as absolute numbers and percentages (%), and continuous variables are reported as medians with [interquartile range]. The statistical significance threshold was set at 0.05. Significant *p*-values are shown **in bold**. Abbreviations: EHR = endemic or hyperendemic region; NIC = non-immunocompromised; IS = immunosuppressive; IC = immunocompromised.

**Table 2:** Comorbidities, history of strongyloidiasis, substance use and immunosuppressive factors in WP and P groups.

Characteristics	Total (%)	WP (%)	P (%)	*p*-Value
	n = 358	n = 225	n = 133	
**Comorbidities**				
AHT	112 (31.3)	59 (26.2)	53 (39.8)	**0.0093**
Diabetes	54 (15.1)	28 (12.4)	26 (19.5)	0.0920
Obesity	43 (12.0)	29 (12.9)	14 (10.5)	0.6144
Hepatic steatosis or cirrhosis	42 (11.7)	27 (12.0)	15 (11.3)	0.8671
Neoplasia	35 (9.8)	23 (10.2)	12 (9.0)	0.8542
Asthma	30 (8.4)	19 (8.4)	11 (8.3)	1
CRF	27 (7.5)	18 (8.0)	9 (6.8)	0.8364
HF	16 (4.5)	14 (6.2)	2 (1.5)	**0.0372**
COPD	7 (2.0)	4 (1.8)	3 (2.3)	0.7139
CID				
HIV +	27 (7.5)	15 (6.7)	11 (8.3)	0.6740
Chronic viral hepatitis	26 (7.3)	13 (5.8)	14 (10.5)	0.1456
≥1 CID	49 (13.7)	25 (11.1)	24 (18.0)	0.0798
Autoimmune disease				
Rheumatic	27 (7.5)	17 (7.6)	10 (7.5)	1
Digestive	11 (3.1)	8 (3.6)	3 (2.3)	0.7527
Neurological	5 (1.4)	3 (1.3)	2 (1.5)	1
Dermatological	5 (1.4)	3 (1.3)	2 (1.5)	1
Other	3 (0.8)	3 (1.3)	0 (0.0)	0.2976
≥1 autoimmune disease	47 (13.1)	31 (13.8)	16 (12.0)	0.7465
≥1 comorbidity	217 (60.6)	123 (54.7)	94 (70.7)	**0.0035**
**History of strongyloidiasis**	22 (6.1)	20 (8.9)	2 (1.5)	**0.0051**
Treatment received	16 (4.5)	14 (6.2)	2 (1.5)	**0.0372**
**Substance use**				
Alcohol	87 (24.3)	53 (23.6)	34 (25.6)	0.7028
Tobacco	84 (23.5)	57 (25.3)	27 (20.3)	0.3036
**IFs**				
Corticosteroids	27 (7.5)	18 (8.0)	9 (6.8)	0.8364
Other immunosuppressants	15 (4.2)	8 (3.6)	7 (5.3)	0.4286
Hematological disease	13 (3.6)	8 (3.6)	5 (3.8)	1
Chemotherapy	8 (2.2)	5 (2.2)	3 (2.3)	1
HIV + et CD4 < 200/µL	6 (1.7)	5 (2.2)	1 (0.8)	0.4183
Solid organ transplantation	4 (1.1)	3 (1.3)	1 (0.8)	1
≥1 IF	52 (14.5)	36 (16.0)	16 (12.0)	0.3531

Qualitative variables are expressed as absolute numbers and percentages (%). The statistical significance threshold was set at 0.05. Significant *p*-values are shown **in bold.** Abbreviations: AHT = arterial hypertension; CRF = chronic renal failure; HF = heart failure; COPD = chronic obstructive pulmonary disease; CID = chronic infectious diseases; HIV + = seropositivity for the human immunodeficiency virus; IFs = immunosuppressive factors; CD4 = CD4 T lymphocytes.

**Table 3:** (**a**). Comparison of outcome and severe complications between the untreated (NT) and treated (T) groups. (**b**). Analysis of the appropriateness of *S. stercoralis* management in the weakly positive (WP) and positive (P) groups.

**(a)**				
Characteristics	Total (%)	NT (%)	T (%)	*p*-value
	n = 358	n = 161	n = 197	
**Outcome**				
Favorable	244 (68.2)	86 (53.4)	158 (80.2)	**<0.0001**
Patient alive and asymptomatic	222 (62.0)	71 (44.1)	151 (76.6)	**<0.0001**
Normalization of eosinophil count	92 (25.7)	16 (9.9)	76 (38.6)	**<0.0001**
Decrease in antibody levels	59 (16.5)	14 (8.7)	45 (22.8)	**0.0003**
Resolution of symptoms	47 (13.1)	5 (10.6)	42 (21.3)	**<0.0001**
Negativation of PSE	2 (0.6)	0 (0.0)	2 (1.0)	0.5037
Unfavorable	40 (11.2)	23 (14.3)	17 (8.6)	0.0951
Persistent eosinophilia	27 (7.5)	15 (9.3)	12 (6.1)	0.3150
Persistent symptoms	15 (4.2)	9 (5.6)	5 (3.0)	0.2918
Deaths potentially linked to *S. stercoralis*	2 (0.6)	2 (1.2)	0 (0.0)	0.2016
Unknown	74 (20.7)	52 (32.3)	22 (11.2)	**<0.0001**
**Severe complications**				
Bacterial infection	11 (3.1)	7 (4.3)	4 (2.0)	0.2321
Positive blood culture	4 (1.1)	3 (1.9)	1 (0.5)	0.3303
Organ failure	6 (1.7)	2 (1.2)	4 (2.0)	0.6944
**(b)**				
**Characteristics**	Total (%)	WP (%)	P (%)	*p*-value
	n = 358	n = 225	n = 133	
**Appropriate management**	51 (14.2)	18 (8.0)	33 (24.8)	**<0.0001**
**Unmet criteria**				
No PSE	118 (33.0)	79 (35.1)	39 (29.3)	0.2965
No treatment	161 (45.0)	127 (56.4)	34 (25.6)	**<0.0001**
No RT in IC patients	44 (12.3)	34 (15.1)	10 (7.5)	**0.0447**
No RT with persistent symptoms	14 (3.9)	10 (4.4)	4 (3.0)	0.5834
No serological follow-up	273 (76.3)	185 (82.2)	88 (66.2)	**0.0008**
No eosinophil count monitoring	36 (10.1)	19 (8.4)	17 (12.8)	0.2054

Qualitative variables are expressed as absolute numbers and percentages (%). The statistical significance threshold was set at 0.05. Significant *p*-values are shown **in bold**. Abbreviations: PSE = parasitological stool examination; RT = repeated treatment; IC = immunocompromised.

## Data Availability

The original contributions presented in this study are included in the article. Further inquiries can be directed to the corresponding author.

## Abbreviations

The following abbreviations are used in this manuscript:

WHO	World Health Organization
AHT	Arterial hypertension
CID	Chronic infectious diseases
CHU-St-Pierre	Centre Hospitalier Universitaire Saint-Pierre
COPD	Chronic Obstructive Pulmonary Disease
CRF	Chronic Renal Failure
DB	Digestive biopsies
EHR	Endemic or Hyperendemic region
EA	Endoscopic anomalies
ELISA	Enzyme-linked immunosorbent assay
HF	Heart failure
HIS	Hyperinfection syndrome
HIV	Human immunodeficiency virus
HUB-Erasme	Hôpital Universitaire de Bruxelles-Erasme
IF	Immunosuppressive factors
NT	Untreated group
P	Positive
PE	Parasitological exam
PSE	Parasitological stool examination
RT	Repeated treatment
*S. stercoralis*	*Strongyloides stercoralis*
T	Treated group
TD	Treatment delay
WP	Weakly positive

## Appendix A. Details of the ELISA Kits Used

Serological diagnosis was performed using two different ELISAs during the study period. From January 2012 to November 2012, the parasitology laboratory of HUB-Erasme used an in-house ELISA assay based on a *Strongyloides ratti* antigen with a serum dilution of 1:400. From November 2012 onwards, the commercial SCIMEDX STRONGY-96 ELISA kit (SciMedx Corporation, Denville, NJ, USA), also based on a *Strongyloides ratti* antigen, was used with a serum dilution of 1:64. This kit was used at HUB-Erasme from November 2012 to December 2021 and at CHU Saint-Pierre throughout the study period.

For each tested sample, the serological result was expressed as an index *i* (%), calculated from the absorbance values of the sample (As), negative control (Aneg), and positive control (Apos), according to the following formula:

*i* = [(As − Aneg)/(Apos − Aneg)] × 100.

Results were interpreted as follows: *i* < 20, negative; 20 ≤ *i* < 30, very weakly positive; 30 ≤ *i* < 70, weakly positive; and *i* ≥ 70, positive.

## Appendix B. Details Regarding Data Collection and Definitions

The term “ethnicity” has been used to designate the following phenotypic groups: Caucasian, Sub-Saharan African, North African, Hispanic and Asian.

The considered reasons for serological testing were: signs and symptoms suggestive of strongyloidiasis, blood eosinophilia, screening (for non-immunocompromised patients in the context of return from travel or childhood in an HER, and for immunocompromised patients or those who will be immunosuppressed in the near future), a history of parasitosis, and “other reason”.

Several comorbidities and IFs were identified, but only those identified before the serodiagnosis of strongyloidiasis were reported. The IFs collected were solid organ transplantation, hematological diseases, HIV+ status with a CD4 lymphocyte count < 200/μL, recent chemotherapy (<6 months) and immunosuppressive treatments ongoing at the time of serology (corticoids or other immunosuppressive drugs).

For the clinical presentation, we indicated whether each patient was symptomatic or not for *S. stercoralis*. We registered the following symptoms (with their date of onset): general, gastrointestinal, respiratory, dermatological, and “other symptoms”. Any patient presenting with one of these complaints was considered symptomatic, provided it could not be explained by another concomitant pathology. Finally, we noted and specified the physical examination abnormalities attributed to *S. stercoralis* (cutaneous, abdominal or “other”). In symptomatic individuals, we searched for potential HIS.

Laboratory findings reported were the presence of blood eosinophilia (defined as an absolute eosinophil count >500/µL) and the first date on which it was identified, positive parasitic serologies for other helminths and protozoa, parasitological stool examination (PSE), parasitological examination (PE) of urine and other body fluids, tissue biopsies and blood cultures.

Three treatment categories were identified: treatment unknown (no treatment documented in the file or patient lost to follow-up), no treatment (decision not to treat) and treatment administered. We recorded the date of administration of the first dose, the type of drug, and the number of doses prescribed. Repeated treatment was defined as an additional treatment administered more than one month after the initial dose.

Qualitative variables are expressed as absolute numbers and percentages (%). No significant difference was observed between the weakly positive (WP) and positive (P) serological groups (*p* = 0.1533).

**Table A1:** Laboratory tests and digestive endoscopies in patients with weakly positive (WP) and positive (P) *S. stercoralis* serology.

Characteristics	Total (%)	WP (%)	P (%)	*p*-Value
	n = 358	n = 225	n = 133	
**Blood eosinophilia**				
>500/μL (missing data = 6)	160/352 (45.5)	67/222 (30.2)	93/130 (71.5)	**<0.0001**
**Serology for other parasites**				
Performed	319/358 (89.1)	196/225 (87.1)	123/133 (92.5)	0.1594
Positive for helminths	133/319 (41.7)	58/196 (29.6)	75/123 (60.6)	**<0.0001**
Positive for protozoa	33/319 (10.3)	22/196 (11.2)	11/123 (8.9)	0.5749
**PSE**				
Performed	240/358 (67.0)	146/225 (64.9)	94/133 (70.7)	0.2965
Positive for *S. stercoralis*	5/240 (2.1)	3/146 (2.1)	2/94 (2.1)	1
Positive for other parasites	39/240 (16.2)	15/146 (10.3)	24/94 (25.5)	**0.0023**
**PE of urine**				
Performed	19/358 (5.3)	11/225 (4.9)	8/133 (6.0)	0.6345
Positive	0/19 (0.0)	0/11 (0.0)	0/8 (0.0)	/
**PE of other biological fluids**				
Performed	26/358 (7.3)	16/225 (7.1)	10/133 (7.5)	1
Positive	0/26 (0.0)	0/16 (0.0)	0/10 (0.0)	/
**Digestive endoscopies (total)**	136 (38.0)	101 (44.9)	35 (26.3)	**0.0005**
Endoscopies without PSE	46/136 (33.8)	35/101 (34.7)	11/35 (31.4)	0.8367
EA potentially related to *S. stercoralis*	16 (4.5)	11 (4.9)	5 (3.7)	0.7929
**Tissue biopsies**				
Digestive	107/358 (29.9)	81/225 (36.0)	26/133 (19.5)	**0.0012**
Presence of *S. stercoralis*	4/107 (3.7)	1/81 (1.2)	3/26 (11.5)	**0.0437**
Skin	9/358 (2.5)	6/225 (2.7)	3/133 (2.3)	1
Presence of *S. stercoralis*	0/9 (0.0)	0/6 (0.0)	0/3 (0.0)	/
Other	11/358 (3.1)	8/225 (3.6)	3/133 (2.3)	0.7527
Presence of *S. stercoralis*	0/11 (0.0)	0/8 (0.0)	0/3 (0.0)	/

Qualitative variables are expressed as absolute numbers and percentages (%). The statistical significance threshold was set at 0.05. Significant *p*-values are shown **in bold.** Abbreviations: PSE = parasitological stool examination; PE = parasitological examination; EA = endoscopic anomalies.

**Table A2:** Therapeutic management according to serological status in patients with weakly positive (WP) and positive (P) *S. stercoralis* serology.

Characteristics	Total (%)	WP (%)	P (%)	*p*-Value
	n = 358	n = 225	n = 133	
**Treatment**				
Administered	197 (55.0)	98 (43.6)	99 (74.4)	**<0.0001**
Unknown	58 (16.2)	36 (16.0)	22 (16.5)	0.8832
**Molecule (among treated patients)**	n =197	n = 98	n = 99	
Ivermectin	173 (87.8)	83 (84.7)	90 (90.9)	0.1985
Mebendazole	11 (5.6)	6 (6.1)	5 (5.1)	0.7673
Albendazole	9 (4.6)	6 (6.1)	3 (3.0)	0.3310
Other	4 (2.0)	3 (3.1)	1 (1.0)	0.3686
**Number of ivermectin doses** **(among ivermectin-treated patients)**	n = 173	n = 83	n = 90	
1 dose	64 (37.0)	36 (43.4)	28 (31.1)	0.1154
2 doses	58 (33.5)	26 (31.3)	32 (35.6)	0.6295
≥3 doses	14 (8.1)	4 (4.8)	10 (11.1)	0.1667
Unknown	37 (21.4)	17 (20.5)	20 (22.2)	0.8537

Treatment administration was reported using the total cohort as denominator. Drug distribution was calculated among treated patients, and the number of ivermectin doses among ivermectin-treated patients. Qualitative variables are expressed as absolute numbers and percentages (%). The statistical significance threshold was set at 0.05. Significant *p*-values are shown in **bold**.
